# A systematic review of the prevalence of mental disorders, cognitive impairment and dementia amongst older adults populace in Egypt

**DOI:** 10.1192/bjo.2021.760

**Published:** 2021-06-18

**Authors:** Noha Sabry, George Tadros, Opeyemi Odejimi

**Affiliations:** 1 Cairo University; 2 Aston University, Birmingham and Solihull Mental Health NHS Foundation Trust; 3 Birmingham and Solihull Mental Health NHS Foundation Trust

## Abstract

**Aims:**

This study aims to review current evidence of the prevalence of mental disorders amongst the elderly populace in Egypt. This will be achieved by estimating the current prevalence and then identifying any sociodemographic correlates with mental disorders.

**Background:**

Mental disorders are the leading cause of disabilities amongst the older adult populace worldwide. The population of the older adult in Egypt is fast growing. According to the Egypt, latest national census in 2014, the population of individuals aged 60 years and above is 6.9% and this is expected to nearly double by the year 2031, with a projection of 11.5% forecasted. In fact, it has been estimated that cost per person of mental health diseases such as dementia in low-income countries is approximately £686 ($868) and £2456 ($3109) in lower-middle income countries like Egypt.

**Method:**

Electronic search of five key databases (MEDLINE, PsychINFO, EMBASE, AMED and PubMed) was carried from their date of inception. In addition, reference list scanning, key journal searching, citation searching and relevant internet resources were conducted. Papers were included, if they were published in English, point prevalence studies carried out on older adults Egyptians aged 60 years and above. In addition, mental disorders, cognitive impairment or dementia had to be ascertained using any validated diagnostic tools. Studies which did not meet any of the criteria detailed above were excluded.

**Result:**

16 studies were included in this review. Four main mental disorders were identified, these are: depression, anxiety, cognitive impairment and dementia. Reported prevalence of Depression, anxiety, dementia and cognitive impairment are 23.7-74.5%, 14.2-72%, 3.66-39.2%, and 1.74 to 51.4% respectively. Anxiety and depression was positively correlated with female gender, increasing age and lower educational status. Also, cognitive impairment and dementia was positively correlated with age, illiteracy or low education. However, there appears to be inconsistencies in the diagnostic tools used.

**Conclusion:**

This research brings to the forefront the scale of mental disorders amongst the elderly in Egypt. This may help ensure evidence-based initiatives are put in place and also priority is given to resource allocation for geriatric mental disorders in Egypt.

